# Case report: *Sarcocystis speeri, Aspergillus fumigatus*, and novel *Treponema* sp. infections in an adult Atlantic spotted dolphin (*Stenella frontalis*)

**DOI:** 10.3389/fvets.2023.1132161

**Published:** 2023-04-03

**Authors:** Sarah Emily Balik, Robert James Ossiboff, Nicole Indra Stacy, James F. X. Wellehan, Elodie E. Huguet, Aitor Gallastegui, April L. Childress, Brittany E. Baldrica, Brittany A. Dolan, Laurie E. Adler, Michael Thomas Walsh

**Affiliations:** ^1^Department of Large Animal Clinical Sciences, College of Veterinary Medicine, University of Florida, Gainesville, FL, United States; ^2^Department of Comparative, Diagnostic, and Population Medicine, College of Veterinary Medicine, University of Florida, Gainesville, FL, United States; ^3^Department of Small Animal Clinical Sciences, College of Veterinary Medicine, University of Florida, Gainesville, FL, United States; ^4^Emerald Coast Wildlife Refuge, Navarre, FL, United States

**Keywords:** apicomplexa, case report, cetacean·marine mammal, encephalitis, glossitis, pneumonia, spirochaetaceae, trichocomaceae

## Abstract

A complete postmortem examination, including a computed tomography scan “virtopsy” (virtual necropsy), gross necropsy, cytology, histology, and molecular diagnostics were performed to investigate the cause of death of a deceased adult male Atlantic spotted dolphin (*Stenella frontalis*) that stranded on Pensacola Beach, Florida, USA in February 2020. Significant findings included chronic inflammation of the meninges, brain, and spinal cord with intralesional protozoa (identified as *Sarcocystis speeri via* 18S rRNA and ITS-1 sequences), suppurative fungal tracheitis and bronchopneumonia (identified as *Aspergillus fumigatus via* ITS-2 gene sequence) and ulcerative bacterial glossitis (associated with a novel *Treponema* species, *Candidatus* Treponema stenella, identified *via* 23S rRNA gene sequence). This is the first reported case of *S. speeri* in a marine mammal. Little is understood about the epidemiology of *S. speeri*, including the identity of its intermediate hosts. The findings of this case suggest that *S. frontalis* may be a capable aberrant host and experience morbidity and mortality from this parasite. It is suspected that the novel *Treponema* and *Aspergillus fumigatus* infections were opportunistic or secondary to immunosuppression, either due to *S. speeri* infection or other co-morbidities.

## 1. Introduction

Atlantic spotted dolphins (*Stenella frontalis*) are a relatively abundant species of dolphin, endemic to the Atlantic Ocean waters of West Africa, the Caribbean, South America, and the southeastern USA (1, 2). Some subpopulations of *S. frontalis* are pelagic, but other subpopulations native to the southeastern USA and the Bahamas are usually found in shallower, coastal waters (1). Causes of morbidity and mortality previously reported for this species include fishing interactions, infections such as *Erysipelothrix rhusiopathiae* and *Aspergillus fumigatus*, as well as cases of neoplastic diseases (1, 3–7).

*Aspergillus* species are ubiquitous fungi, commonly reported as causes of fungal infections in marine mammals but are often opportunistic or secondary to systemic disease such as cetacean morbillivirus (5, 8, 9). There is one report of an *Aspergillus fumigatus* bronchopneumonia associated mortality in a *S. frontalis* in Brazil (5).

*Sarcocystis* species are apicomplexan protozoan parasites that have obligate two-host life cycles. Sexual reproduction (gametogony) occurs only in the definitive host and asexual reproduction (schizogony) occurs in the intermediate host. There is typically greater specificity for definitive hosts. *Sarcocystis* spp. have been reported in all groups of marine mammals except for sirenians (10). Sarcocysts may be considered incidental findings in the skeletal muscles of marine mammals, due to exposure to feces from terrestrial definitive hosts (11–15). Infection of the central nervous system is more likely to be clinically significant. *Sarcocystis speeri* is closely related to *S. neurona*, and opossums in the genus *Didelphis* are the definitive hosts for both species (16, 17). The epidemiology and intermediate hosts of *S. speeri* are largely unknown, although there is a recent report of *S. speeri* found in the brain and heart of a blue-fronted Amazon parrot (*Amazona aestiva*) (18). Unspeciated intramuscular sarcocysts have been identified incidentally in *S. frontalis* in the Canary Islands (11). There is also a recent report of meningoencephalitis associated with an unidentified *Sarcocystis*-like organism in two striped dolphins (*Stenella coeruleoalba*) off the Ligurian coast of Italy (12).

*Treponema* species are bacteria in the phylum Spirochaetes well-characterized for their role as pathogens. *Treponema* spp. have been identified as the causative agents in many animal diseases, including digital dermatitis in cattle and sheep and periodontal disease in humans and dogs (19–26). However, some *Treponema* spp. are considered commensal in animals ranging from termites to cattle (21, 26). *Treponema* spp. have been characterized as part of the free-ranging Atlantic bottlenose dolphin (*Tursiops truncatus*) microbiome, but association with disease has not been reported to date (27).

The case report herein describes a stranded cetacean from the Western hemisphere infected with *Sarcocystis* sp. cysts in the central nervous system, which were identified as *S. speeri via* polymerase chain reaction (PCR) and Sanger sequencing. This animal also had ulcerative glossitis associated with a novel *Treponema* species (*Candidatus* Treponema stenella), also identified by PCR and subsequent sequencing.

## 2. Case description

An adult male *S. frontalis* stranded alive on Pensacola Beach, Florida, USA on the 24th of February 2020. Members of the public made multiple unauthorized attempts to push the animal back into the Gulf of Mexico. The animal was found deceased in the water later that day. The carcass was recovered and transported in sternal position packed in ice to the University of Florida College of Veterinary Medicine for postmortem examination.

Prior to necropsy, a postmortem computed tomography scan (CT), or “virtopsy,” was performed on the whole body using a Toshiba Aquilion Prime 160-slice helical scanner (Cannon Medical Systems, Tustin, CA, USA) (8). The images were reconstructed into 0.5-mm thick overlapping slices in transverse, sagittal, and dorsal planes with soft tissue, lung, and bone algorithms. Images were reviewed on two commercially available picture archiving and communications systems: Merge PACS^TM^ (IBM Watson Healthcare, Cambridge, MA, USA) and Horos^TM^ (Purview, Annapolis, MD, USA). Three-dimensional reformations of the transverse images were generated using a volumetric display.

**Figure 1:**
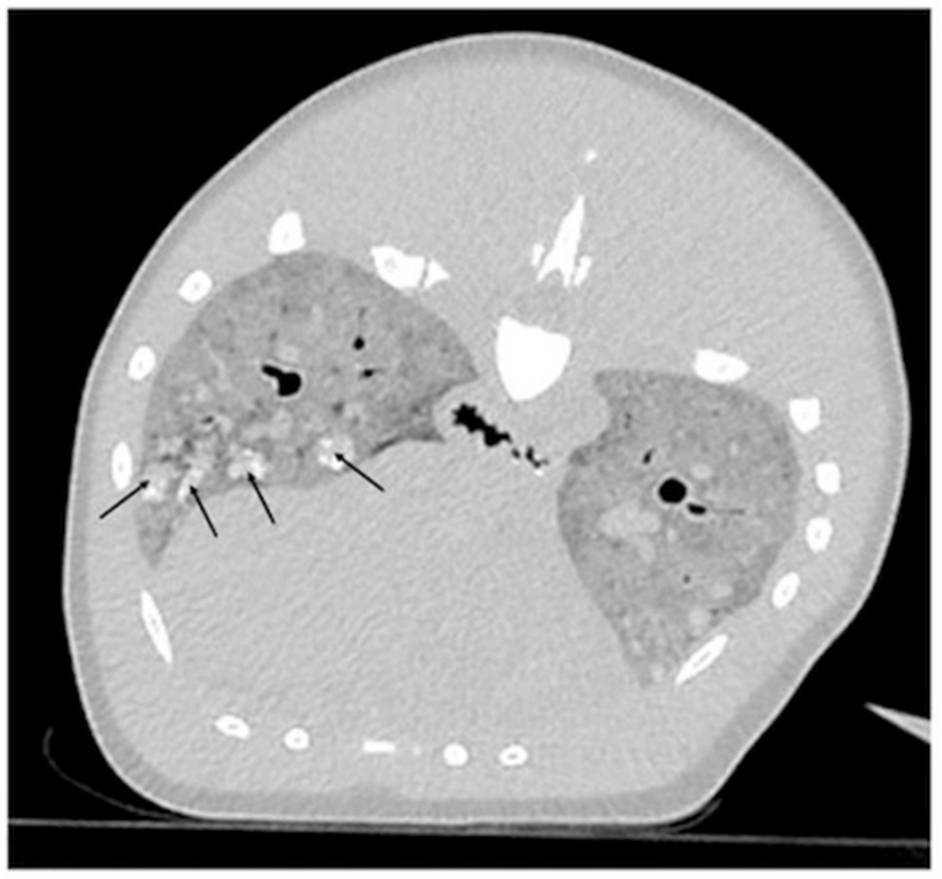
Computed tomographic (CT) transverse image centered on the mid-thorax reconstructed in a lung algorithm of an adult male *Stenella frontalis*. Diffusely, the pulmonary parenchyma has a severe increase in soft tissue attenuation and contains multifocal and ill-defined soft tissue attenuating nodules, some of which have mineral foci (black arrows).

Endotracheal intubation for lung expansion was not performed prior to imaging due to the technical difficulty of intubation in a dolphin of this size. CT images revealed that the pulmonary parenchyma had severely increased soft tissue attenuation bilaterally (Figure 1). The diffuse changes in the pulmonary parenchyma were attributed to postmortem atelectasis. Within the lungs there were numerous small, poorly-defined, rounded soft-tissue attenuating nodules, some with mineral attenuating foci (Figure 1). The nodules measured up to 2 centimeters in diameter. These represented antemortem lesions, and the differential diagnosis included chronic granulomatous pneumonia of fungal, parasitic and/or bacterial etiologies. Visible abnormalities were absent from the central nervous system.

**Figure 2:**
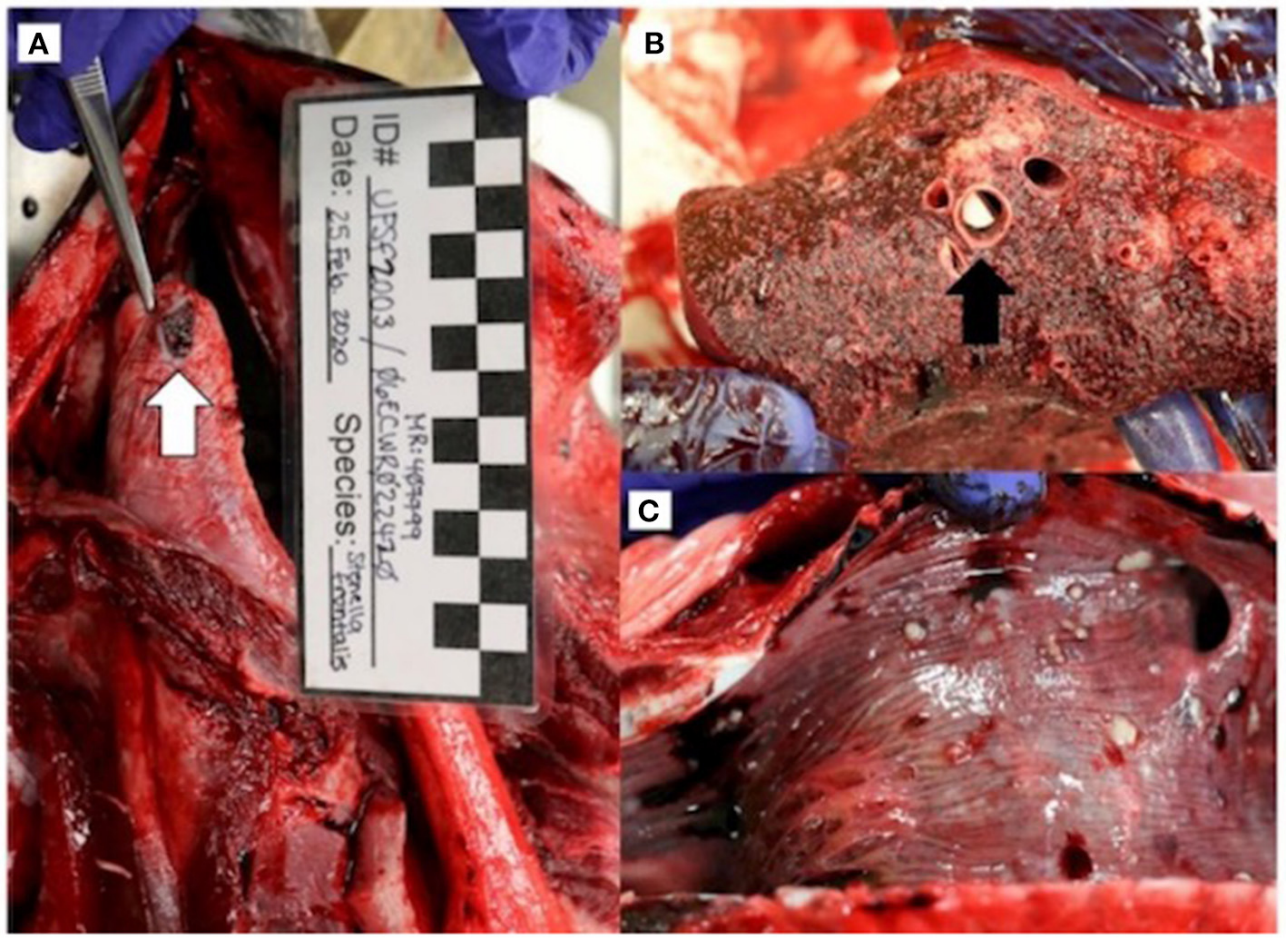
Gross necropsy findings in an adult male *Stenella frontalis* included a 1.0 x 0.8 cm ulcerated lesion (white arrow) on the ventral aspect of the tongue **(A)**, lungs with multifocal to coalescing white raised nodules **(B)** with necrotic debris within bronchi (black arrow), and thick mucus and 1 to 4 mm free floating white debris in the anterior trachea **(C)**.

On gross necropsy, the animal was thin, with decreased nuchal fat. There were no notable external lesions or indications of human interaction present. Prescapular and mesenteric lymphadenopathy was noted. An ulcerated lesion was present on the ventral aspect of the tongue (Figure 2A). The gastrointestinal tract was empty. There were multifocal to coalescing regions of white, palpable nodules in the lungs (Figure 2B) that correlated to the nodules identified on CT, as well as thick mucus and white material in the anterior trachea (Figure 2C) and secondary bronchi. There were no gross lesions noted in the brain or spinal cord.

**Figure 3:**
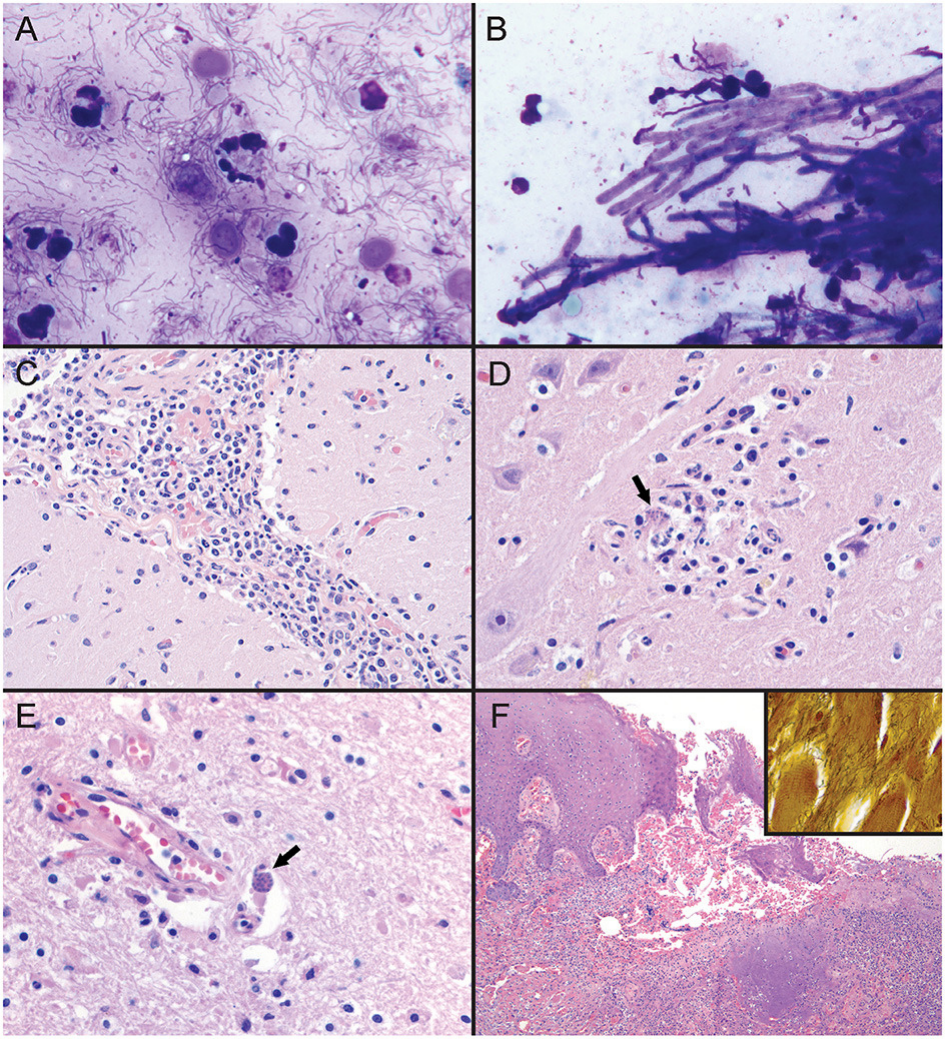
Tissue sections and imprint cytology from an adult male *Stenella frontalis*. **(A)** Imprint of tongue lesion. Mixed bacterial infection with suppurative inflammation, characterized by extracellular and phagocytosed mixed bacilli, diplococci, and spirilliform bacteria (1000x; hematoxylin and eosin stain [HE]). **(B)** Imprint of tracheal mucosa. There is a dense mat of septate branching fungal hyphae and mixed bacilli (1000x; HE). PCR on frozen lung tissue identified the fungus as *Aspergillus fumigatus*. **(C)** Photomicrograph of leptomeninges in cerebral sulcus. Mixed leukocytes, with a predominant population of lymphocytes and histiocytes expand the leptomeninges (400x; HE). **(D)** Photomicrograph of the right midbrain. Apicomplexan protozoa (black arrow) are present in a focus of malacia and gliosis (600x; HE). **(E)** Photomicrograph of right frontal cortex. Apicomplexan bradyzoites (black arrow) are adjacent to a vessel in the gray matter. Increased numbers of glial cells are scattered throughout the neuroparenchyma (600x; hematoxylin and eosin stain). PCR on frozen central nervous system tissue identified *Sarcocystis speeri* within the samples. **(F)** Photomicrograph of tongue lesion. There is regional ulceration of the glossal mucosa with a underlying bed of cellular and necrotic debris, and intact and degenerate neutrophils. HE; 100x. [Inset] Large numbers of spirilliform bacteria are present throughout the ulcerative lesion (600x; Warthin-Starry stain). PCR on frozen tongue tissue identified the bacteria as a *Treponema* sp.

Cytologic findings based on tissue imprints stained with Wright-Giemsa (Harleco^®^ EMD Millipore, Billerica, MA, USA) included reactive hyperplasia of the prescapular lymph node, and suppurative inflammation with squamous cell hyperplasia and mixed bacterial infection of the tongue lesion with abundant extracellular and phagocytized bacilli, diplococci, and spirilliform bacilli (Figure 3A). The tracheal mucosa had suppurative inflammation with respiratory epithelial cell hyperplasia, mixed bacilli, and dense mats of branching fungal hyphae (Figure 3B). No cytologic abnormalities were noted on evaluation of direct smears of cerebrospinal fluid. Gastric fluid direct smear showed suppurative inflammation with epithelial cell hyperplasia and mixed bacteria.

Histologically, using standard hematoxylin and eosin staining methods, there was severe, chronic-active, suppurative bronchopneumonia with bronchiectasis and associated mixed bacilli and fungal hyphae, as well as chronic, lymphohistiocytic, meningoencephalomyelitis and gliosis with protozoa and rare protozoal cysts (Figures 3C–E). Marked chronic-active suppurative glossitis with ulceration and mixed bacteria, moderate lingual sialadenitis, and severe chronic-active suppurative prostatitis were identified, but considered of questionable significance regarding their role as contributing factors to cause of death (Figure 3F). Warthin-Starry staining of the tongue lesion highlighted the spirilliform bacteria noted on cytology that were not identifiable on routine hematoxylin and eosin stained sections (Figure 3F inset). These bacteria were associated with the suppurative inflammation noted in the tongue lesion. There were no protozoa, protozoal cysts, or other lesions identified on histologic evaluation of skeletal muscle.

Morbilliviruses were absent *via* PCR of brain and lung tissue (University of Georgia^®^ Veterinary Diagnostic Laboratories, Athens, GA, USA) using previously described methods (28). *Brucella* spp. were absent *via* PCR of cerebrospinal fluid and brain (Veterinary Diagnostic Laboratory at the University of Illinois College of Veterinary Medicine, Urbana, IL, USA) using previously described methods (29). At the time of necropsy, a fungal culture of a lung nodule was performed on potato flake agar and inhibitory mold agar incubated at 25°C for 26 days (University of Florida Veterinary Diagnostic Laboratories, Gainesville, FL, USA), but no fungi were isolated. Nucleic acids were extracted from frozen lung tissue (University of Florida Zoological Medicine Diagnostic Laboratory [UF ZMDxL], Gainesville, FL, USA) using a DNeasy blood and tissue kit (Qiagen, Germantown, MD, USA) following the manufacturer's instructions. PCR amplification of the internal transcribed segment 2 (ITS-2) was performed using previously described methods (30). PCR products, as well as positive (DNA extract from animal case of *Fusarium* sp. infection) and negative (water) controls, were resolved on a 1.5% agarose gel with ethidium bromide and were visualized under ultraviolet light. An amplicon of appropriate size was excised and purified using the QIAquick^®^ Gel Extraction kit (Qiagen) following the manufacturer's instructions. The purified amplicon was bidirectionally, commercially (Genewiz, South Plainfield, NJ, USA) sequenced. Sequences were assembled, edited, analyzed, and compared with sequences previously entered in GenBank by nucleotide basic local alignment search tool (BLASTN) (31, 32). The sequence was 100% identical to *Aspergillus fumigatus* (Supplementary Table S1).

Immunohistochemistry for *Toxoplasma gondii* performed on formalin-fixed paraffin embedded brain tissue was negative (Animal Health Diagnostic Center, Cornell University, Ithaca, NY, USA) utilizing a purified rabbit polyclonal IgG anti-*Toxoplasma gondii* antibody (T8075) according to the manufacturer's instructions (US Biological, Salem, MA, USA). For protozoal identification, nucleic acids were extracted at the UF ZMDxL as above from frozen samples of cerebellum, cerebrum, spinal cord, and cranial nerve. Extracts were tested by conventional PCR using both pan-apicomplexan and *Sarcocystis*-specific assays targeting the 18S rRNA and internal transcribed spacer 1 (ITS-1) regions, respectively, using previously described methods (33, 34). Amplification products were resolved, gel purified, and bidirectionally, commercially sequenced, assembled, edited, and analyzed as above. All sequences were identical to each other, and 100% identical to *Sarcocystis speeri* (Genbank accession KT207458; Supplementary Tables S2, S3). Although *T. gondii* infections has been reported in *S. frontalis*, no evidence of coinfection with *S. speeri* was found in this case (35–37).

For genetic characterization of the glossal spirilliform bacteria, a frozen sample of the tongue lesion was submitted to the UF ZMDxL, nucleic acids were extracted as described above and PCR was performed using primers Spirochaete23SF (5'-TCGACCAGTGAGCTRTTACGCAC-3') and Spirochaete23SR (5'-KTACCAAACYCARYYAAACTCCG-3') to target the 23S rRNA region. Amplicons were resolved and analyzed as above, resulting in a 173 base pair product. This product was compared to others in the GenBank database (National Center for Biotechnology Information, Bethesda, MD, USA) database using the BLASTN tool, and was determined to be a novel *Treponema* species most closely related (97.1% nucleotide identity) to *Treponema vincentii* (Genbank accession CP051197); the next two most closely related *Treponema* included *T. phagedenis* (Genbank accession CP054692) and *T. medium* (Genbank accession CP031393) at 96.5% nucleotide identity. The novel sequence was submitted to GenBank (accession MZ267537) and is hereafter referred to as *Candidatus* Treponema stenella.

**Figure 4:**
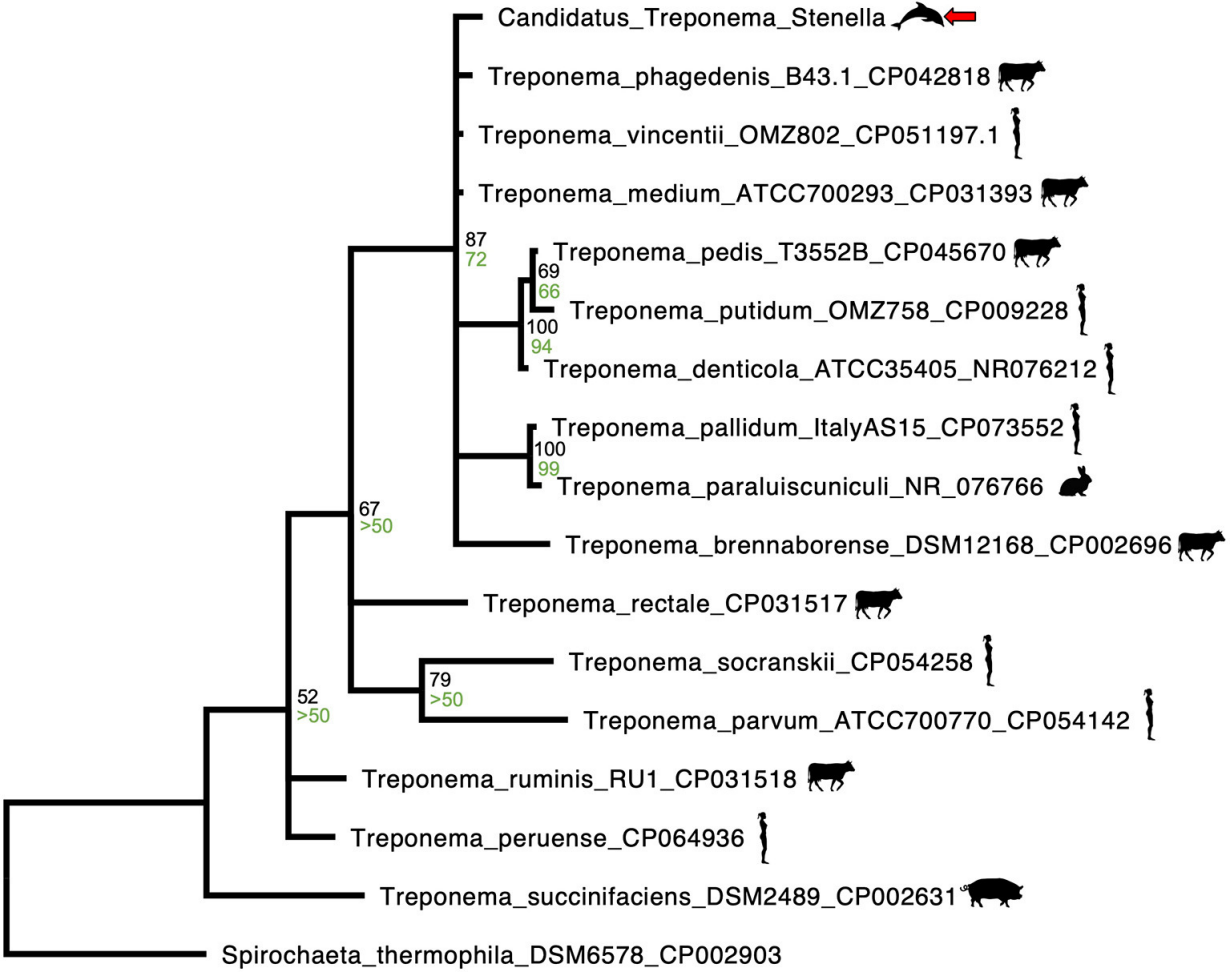
MAFFT alignment Bayesian phylogenetic tree representative of homologous 170 to 173 base pair 23S rRNA sequences of Spirochaetes closely related to a novel *Treponema* species identified from an ulcerative tongue lesion in a *Stenella frontalis*. The outgroup used was *Spirochaeta thermophila* (Genbank accession CP002903). Bayesian posterior probabilities are written in black, above the maximum likelihood bootstrap values, which are written in green. Both posterior probabilities and maximum likelihood bootstrap values were rounded to the nearest whole percent. The novel *Treponema* species (*Candidatus* Treponema stenella) is identified by a red arrow, *Treponema* spp. associated with bovids, humans, pigs or rabbits are identified by accompanying graphics of those species.

Phylogenetic analysis of the novel *Treponema* was performed by first downloading homologous 170 to 173 base pair sequences of 23S rRNA from Spirochaetes closely related to *Candidatus* Treponema stenella were downloaded from GenBank and aligning them using MAFFT (38). Bayesian analysis was then performed with MrBayes on the CIPRES server, utilizing 2,000,000 iterations, a time-reversible model, gamma-distributed proportion of invariant sites and rate of variation (39–41). The outgroup used was *Spirochaeta thermophila* (Genbank accession CP002903). A maximum likelihood analysis of the phylogeny was performed with RAxML on the CIPRES server, utilizing a time-reversible model, gamma-distributed proportion of invariant sites and rate of variation, 1,000 resamplings for bootstrap analysis, and *Spirochaeta thermophila* as the outgroup (42, 43). Maximum likelihood bootstrap values are shown on the Bayesian tree (Figure 4).

## 3. Discussion and conclusions

This is the first report of *S. speeri* infection in the central nervous system of *S. frontalis*, as well as the first documented infection of *S. speeri* in any marine mammal (5, 11, 44). An unknown *Sarcocystis* species has been reported as an incidental finding in two *S. frontalis* that stranded on the Canary Islands, consistent with postulations that dolphins may act as intermediate hosts for *Sarcocystis* parasites (10, 11). No sarcocysts were identified in the skeletal muscle in the present case, and in contrast with the previous report, the finding of *S. speeri* along with inflammation in the central nervous system suggests not only that *S. speeri* was a cause for morbidity, but also that dolphins may be aberrant intermediate hosts for these parasites, similar to *S. canis* hepatitis in sea lions (14, 16, 44). It is possible that previous cases of *S. speeri* have been overlooked in marine mammals due to the genetic analysis required for speciation (17).

Historically, water runoff, sewage, and consumption of benthic invertebrates have been hypothesized to increase the risk for *Sarcocystis* species infection in marine mammals (13, 14). This may put *S. frontalis* that inhabit shallower coastal waters, such as in the Gulf of Mexico, at increased risk (1). *S. frontalis* eat both fishes and benthic invertebrates throughout their range, so pelagic subpopulations could also be at risk if benthic invertebrates are capable transport hosts for *S. speeri* (1, 13).

As *Morbillivirus* and *Brucella* infections were not detected in this case, it is possible that *S. speeri* infection may have led to immunosuppression and secondary *Aspergillus fumigatus* pneumonia, or alternatively, though considered less likely, another unidentified systemic disease or stressor may have caused susceptibility to *S. speeri* and fungal pneumonia. *A. fumigatus* bronchopneumonia was reported as the primary cause of death in the only previous report of *A. fumigatus* in a *S. frontalis* (5). *S. frontalis* may be susceptible to severe disease from pulmonary aspergillosis.

Magnetic resonance imaging (MRI) is a more sensitive imaging modality for identification of central nervous system lesions than CT in cetaceans and other animals, though time, accessibility, and additional expense can influence its use (45). However, it is possible that central nervous system abnormalities would not have been identified in this case if an MRI was performed because intravenous contrast enhancing agents cannot be used postmortem. Although “virtopsy” is an emerging tool to help locate lesions and guide necropsy sample collection, this case demonstrates the importance of conducting complete histopathologic investigations, in concert with saving representative frozen tissue samples, even in the absence of visible lesions (8).

The sequence of the spirochete found in the tongue of this dolphin was most closely related to *Treponema* spp., with *T. vincentii* having the highest sequence identity at 97.1%. Differences in this region of the 23S gene of <1% are observed in the closely related *T. vincentii, T. phagedenis*, and *T. denticola*. As such, it is likely that the spirochete identified in the tongue of this dolphin represents a novel species, *Candidatus* Treponema stenella. The Bayesian phylogenetic analysis performed (Figure 4) revealed close relatedness of *Candidatus* Treponema stenella to multiple *Treponema* species that have been previously associated with either periodontitis in humans (*T. medium, T. putidum, T. denticola*, and *T. parvum*) or digital dermatitis in cattle and sheep (*T. phagedenis* and *T. phagedenis*-like, *T. brennaborense*, and *T. pedis*) (21–26). These two groups do not cluster separately, consistent with previous reports of overlap (e.g., *T. medium* has been reported in periodontal abscesses in humans, and *T. medium* and *T. medium-*like species have been reported in bovine and ovine digital dermatitis) (21–24). This is interesting, given cetaceans' phylogenetic relationship to ruminants (12). A study of dental disease in odontocetes found that *S. frontalis* had a comparatively high rate of dental disease (46).

There is evidence that certain treponemes may be more pathogenic than others, and complex relationships exist between *Treponema* spp. and their hosts (21, 26). For example, symbiotic or commensal *Treponema* spp. act as pathogens in periodontal disease in humans and digital dermatitis (21, 26). It is possible that *Candidatus* T. stenella may be a commensal organism that was an opportunistic pathogen in this case with the infection localized to chronic glossitis. *Treponema* spp. have been described as part of the Atlantic bottlenose dolphin microbiome (*Tursiops truncatus*), with significantly higher abundance in the genital region relative to the oral cavity or other body regions (27). Although disease associated with these *Treponema* spp. have not been reported in dolphins, it is noteworthy that one of the two recently identified *Stenella coeruleoalba* cases from Italy with *Sarcocystis*-like meningoencephalitis had ulcerative glossitis, and it is possible that immunosuppression secondary to *Sarcocysti*s and *Sarcocystis*-like infections could put dolphins at risk for opportunistic infections from commensal *Treponema* spp. or other pathogens, though immunocompromise may be multifactorial (12, 27). More research is needed to elucidate the role of *Treponema* spp. as oral pathogens in cetaceans, especially because *Treponema* are difficult to culture, leaving many uncharacterized and poorly understood (19, 26). Thus, this case highlights the importance of non-culture based methods for identification and characterization of potential pathogens, and also raises concern that *Treponema* spp. (either pathogenic or commensal flora) may have the potential to cause clinically relevant disease in *S. frontalis* or other dolphins that are immunocompromised due to concurrent morbidities.

Further investigation is required to understand the epidemiology, clinical relevance, and pathologic significance of *S. speeri* as an emerging disease in *S. frontalis* and other marine mammals. Careful handling and thorough postmortem diagnostic work up (including imaging, necropsy, tissue fixation, cytology, histology, and collection of frozen tissues for molecular diagnostics) may increase the potential for discovering clinically significant findings in stranded marine animals.

## Data availability statement

The original contributions presented in the study are included in the article/Supplementary material, further inquiries can be directed to the corresponding author.

## Ethics statement

Ethical review and approval was not required for this study because rescue activities and diagnostic investigation were performed under a stranding agreement between National Marine Fisheries Service and the University of Florida's Marine Animal Rescue, and in accordance with general animal welfare standards.

## Author contributions

SB and MW contributed to the pursuit and interpretation of diagnostic work up. EH and AG provided interpretation of the CT scan. RO contributed to preparation and interpretation of histopathology samples. AC performed molecular diagnostics (PCR). RO and JW contributed to preparation, design, and interpretation of molecular diagnostics. JW and SB contributed to the creation of the *Candidatus* Treponema stenella phylogenetic tree. NS conducted preparation and interpretation of clinical pathology samples. BB, LA, and MW participated in carcass recovery, animal transport, and performed the initial necropsy. BD managed submission of tissue samples. All authors contributed to manuscript writing and editing.
