# Supplementary material for: Case report: Sarcocystis speeri, Aspergillus fumigatus, and novel Treponema sp. infections in an adult Atlantic spotted dolphin (Stenella frontalis)
Source: Front Vet Sci. 2023 Apr 3;10:1132161. doi: 10.3389/fvets.2023.1132161 (PMC10106728; doi:10.3389/fvets.2023.1132161)
Supplement: Supplementary file 1 [file Table_1.docx]

| \| Description \| Accession \| Query Coverage \| % identity \| \| --- \| --- \| --- \| --- \| \| Aspergillus fumigatus isolate 933 \| [OQ296938.1](https://www.ncbi.nlm.nih.gov/nucleotide/OQ296938.1?report=genbank&log$=nucltop&blast_rank=1&RID=XURMFDR7016) \| 100% \| 100 \| \| Aspergillus fumigatus isolate 921 \| [OQ296935.1](https://www.ncbi.nlm.nih.gov/nucleotide/OQ296935.1?report=genbank&log$=nucltop&blast_rank=2&RID=XURMFDR7016) \| 100% \| 100 \| \| Aspergillus fumigatus strain ColD Pav_10 \| [OQ248216.1](https://www.ncbi.nlm.nih.gov/nucleotide/OQ248216.1?report=genbank&log$=nucltop&blast_rank=3&RID=XURMFDR7016) \| 100% \| 100 \| \| Aspergillus fumigatus isolate Soil.Fungus_M07 \| [OQ248145.1](https://www.ncbi.nlm.nih.gov/nucleotide/OQ248145.1?report=genbank&log$=nucltop&blast_rank=4&RID=XURMFDR7016) \| 100% \| 100 \| \| Aspergillus fumigatus isolate 0338 \| [OQ244321.1](https://www.ncbi.nlm.nih.gov/nucleotide/OQ244321.1?report=genbank&log$=nucltop&blast_rank=5&RID=XURMFDR7016) \| 100% \| 100 \| \| Aspergillus fumigatus isolate EG-RE-ZnP-nps \| [OQ152057.1](https://www.ncbi.nlm.nih.gov/nucleotide/OQ152057.1?report=genbank&log$=nucltop&blast_rank=6&RID=XURMFDR7016) \| 100% \| 100 \| \| Aspergillus sp. isolate APG-2 \| [OQ073729.1](https://www.ncbi.nlm.nih.gov/nucleotide/OQ073729.1?report=genbank&log$=nucltop&blast_rank=7&RID=XURMFDR7016) \| 100% \| 100 \| \| Aspergillus fumigatus isolate nsm37 \| [OP991939.1](https://www.ncbi.nlm.nih.gov/nucleotide/OP991939.1?report=genbank&log$=nucltop&blast_rank=8&RID=XURMFDR7016) \| 100% \| 100 \| \| Aspergillus fumigatus isolate SJ-21 \| [OP863036.1](https://www.ncbi.nlm.nih.gov/nucleotide/OP863036.1?report=genbank&log$=nucltop&blast_rank=9&RID=XURMFDR7016) \| 100% \| 100 \| \| Aspergillus fumigatus strain p-1222 \| [OP854884.1](https://www.ncbi.nlm.nih.gov/nucleotide/OP854884.1?report=genbank&log$=nucltop&blast_rank=10&RID=XURMFDR7016) \| 100% \| 100 \| \| Aspergillus fumigatus strain F6430 \| [OP001781.1](https://www.ncbi.nlm.nih.gov/nucleotide/OP001781.1?report=genbank&log$=nucltop&blast_rank=11&RID=XURMFDR7016) \| 100% \| 100 \| \| Aspergillus fumigatus isolate VC21 \| [OP782302.1](https://www.ncbi.nlm.nih.gov/nucleotide/OP782302.1?report=genbank&log$=nucltop&blast_rank=12&RID=XURMFDR7016) \| 100% \| 100 \| \| Aspergillus fumigatus isolate VC79 \| [OP782301.1](https://www.ncbi.nlm.nih.gov/nucleotide/OP782301.1?report=genbank&log$=nucltop&blast_rank=13&RID=XURMFDR7016) \| 100% \| 100 \| \| Aspergillus fumigatus isolate VC226 \| [OP782036.1](https://www.ncbi.nlm.nih.gov/nucleotide/OP782036.1?report=genbank&log$=nucltop&blast_rank=14&RID=XURMFDR7016) \| 100% \| 100 \| \| Aspergillus fumigatus isolate C63 \| [OP782035.1](https://www.ncbi.nlm.nih.gov/nucleotide/OP782035.1?report=genbank&log$=nucltop&blast_rank=15&RID=XURMFDR7016) \| 100% \| 100 \| \| Aspergillus fumigatus strain CCPMBF003 \| [OP730546.1](https://www.ncbi.nlm.nih.gov/nucleotide/OP730546.1?report=genbank&log$=nucltop&blast_rank=16&RID=XURMFDR7016) \| 100% \| 100 \| \| Aspergillus fumigatus isolate Af31-22A \| [OP718622.1](https://www.ncbi.nlm.nih.gov/nucleotide/OP718622.1?report=genbank&log$=nucltop&blast_rank=17&RID=XURMFDR7016) \| 100% \| 100 \| \| Aspergillus fumigatus strain P12 \| [OP681422.1](https://www.ncbi.nlm.nih.gov/nucleotide/OP681422.1?report=genbank&log$=nucltop&blast_rank=18&RID=XURMFDR7016) \| 100% \| 100 \| \| Aspergillus fumigatus strain P3 \| [OP681417.1](https://www.ncbi.nlm.nih.gov/nucleotide/OP681417.1?report=genbank&log$=nucltop&blast_rank=19&RID=XURMFDR7016) \| 100% \| 100 \| \| Fungal sp. isolate Contig0013 \| [OP679893.1](https://www.ncbi.nlm.nih.gov/nucleotide/OP679893.1?report=genbank&log$=nucltop&blast_rank=20&RID=XURMFDR7016) \| 100% \| 100 \| \| Aspergillus fumigatus isolate Y.N.161.wers. \| [OP651035.1](https://www.ncbi.nlm.nih.gov/nucleotide/OP651035.1?report=genbank&log$=nucltop&blast_rank=21&RID=XURMFDR7016) \| 100% \| 100 \| \| Aspergillus fumigatus isolate 02436 \| [OP630595.1](https://www.ncbi.nlm.nih.gov/nucleotide/OP630595.1?report=genbank&log$=nucltop&blast_rank=22&RID=XURMFDR7016) \| 100% \| 100 \| \| Aspergillus fumigatus isolate A1f \| [OP630573.1](https://www.ncbi.nlm.nih.gov/nucleotide/OP630573.1?report=genbank&log$=nucltop&blast_rank=23&RID=XURMFDR7016) \| 100% \| 100 \| \| Aspergillus fumigatus strain F9 \| [OP622844.1](https://www.ncbi.nlm.nih.gov/nucleotide/OP622844.1?report=genbank&log$=nucltop&blast_rank=24&RID=XURMFDR7016) \| 100% \| 100 \| \| Aspergillus fumigatus isolate Af7 \| [OP620980.1](https://www.ncbi.nlm.nih.gov/nucleotide/OP620980.1?report=genbank&log$=nucltop&blast_rank=25&RID=XURMFDR7016) \| 100% \| 100 \|   Table S1. BLASTN Results for fungal sequence amplified from frozen lung of an Atlantic spotted dolphin (*Stenella frontalis*) |  |  |  |
| --- | --- | --- | --- | --- | --- | --- | --- | --- | --- | --- | --- | --- | --- | --- | --- | --- | --- | --- | --- | --- | --- | --- | --- | --- | --- | --- | --- | --- | --- | --- | --- | --- | --- | --- | --- | --- | --- | --- | --- | --- | --- | --- | --- | --- | --- | --- | --- | --- | --- | --- | --- | --- | --- | --- | --- | --- | --- | --- | --- | --- | --- | --- | --- | --- | --- | --- | --- | --- | --- | --- | --- | --- | --- | --- | --- | --- | --- | --- | --- | --- | --- | --- | --- | --- | --- | --- | --- | --- | --- | --- | --- | --- | --- | --- | --- | --- | --- | --- | --- | --- | --- | --- | --- | --- | --- | --- | --- |
