# Supplementary material for: Case report: Sarcocystis speeri, Aspergillus fumigatus, and novel Treponema sp. infections in an adult Atlantic spotted dolphin (Stenella frontalis)
Source: Front Vet Sci. 2023 Apr 3;10:1132161. doi: 10.3389/fvets.2023.1132161 (PMC10106728; doi:10.3389/fvets.2023.1132161)
Supplement: Supplementary file 2 [file Table_2.docx]

| Table S2. BLASTN Results for apicomplexan 18S sequence amplified from frozen CNS samples of an Atlantic spotted dolphin (*Stenella frontalis*)   \| Description \| Accession \| Query Coverage \| % identity \| \| --- \| --- \| --- \| --- \| \| Sarcocystis speeri \| [KT207458.1](https://www.ncbi.nlm.nih.gov/nucleotide/KT207458.1?report=genbank&log$=nucltop&blast_rank=1&RID=XUT9F8KR013) \| 100% \| 100 \| \| Sarcocystis cf. falcatula \| [AF389339.1](https://www.ncbi.nlm.nih.gov/nucleotide/AF389339.1?report=genbank&log$=nucltop&blast_rank=2&RID=XUT9F8KR013) \| 100% \| 98.8 \| \| Sarcocystis sp. clone Ma36.4 \| [OL830326.1](https://www.ncbi.nlm.nih.gov/nucleotide/OL830326.1?report=genbank&log$=nucltop&blast_rank=3&RID=XUT9F8KR013) \| 99% \| 98.78 \| \| Sarcocystis sp. clone Ma36.1 \| [OL830324.1](https://www.ncbi.nlm.nih.gov/nucleotide/OL830324.1?report=genbank&log$=nucltop&blast_rank=4&RID=XUT9F8KR013) \| 99% \| 98.78 \| \| Sarcocystis sp. clone Ma29.1 \| [OL830321.1](https://www.ncbi.nlm.nih.gov/nucleotide/OL830321.1?report=genbank&log$=nucltop&blast_rank=5&RID=XUT9F8KR013) \| 99% \| 98.78 \| \| Sarcocystis sp. clone Ma28.3 \| [OL830319.1](https://www.ncbi.nlm.nih.gov/nucleotide/OL830319.1?report=genbank&log$=nucltop&blast_rank=6&RID=XUT9F8KR013) \| 99% \| 98.78 \| \| Sarcocystis sp. clone Ma27.3 \| [OL830318.1](https://www.ncbi.nlm.nih.gov/nucleotide/OL830318.1?report=genbank&log$=nucltop&blast_rank=7&RID=XUT9F8KR013) \| 99% \| 98.78 \| \| Sarcocystis sp. clone Ma27.2 \| [OL830317.1](https://www.ncbi.nlm.nih.gov/nucleotide/OL830317.1?report=genbank&log$=nucltop&blast_rank=8&RID=XUT9F8KR013) \| 99% \| 98.78 \| \| Sarcocystis sp. clone Ma27.1 \| [OL830316.1](https://www.ncbi.nlm.nih.gov/nucleotide/OL830316.1?report=genbank&log$=nucltop&blast_rank=9&RID=XUT9F8KR013) \| 99% \| 98.78 \| \| Sarcocystis sp. clone Ma18.7 \| [OL830315.1](https://www.ncbi.nlm.nih.gov/nucleotide/OL830315.1?report=genbank&log$=nucltop&blast_rank=10&RID=XUT9F8KR013) \| 99% \| 98.78 \| \| Sarcocystis sp. clone Ma12.2 \| [OL830312.1](https://www.ncbi.nlm.nih.gov/nucleotide/OL830312.1?report=genbank&log$=nucltop&blast_rank=11&RID=XUT9F8KR013) \| 99% \| 98.78 \| \| Sarcocystis sp. clone Ma11.3 \| [OL830310.1](https://www.ncbi.nlm.nih.gov/nucleotide/OL830310.1?report=genbank&log$=nucltop&blast_rank=12&RID=XUT9F8KR013) \| 99% \| 98.78 \| \| Sarcocystis sp. clone Ma11.2 \| [OL830309.1](https://www.ncbi.nlm.nih.gov/nucleotide/OL830309.1?report=genbank&log$=nucltop&blast_rank=13&RID=XUT9F8KR013) \| 99% \| 98.78 \| \| Sarcocystis sp. clone Ma6.6 \| [OL830304.1](https://www.ncbi.nlm.nih.gov/nucleotide/OL830304.1?report=genbank&log$=nucltop&blast_rank=14&RID=XUT9F8KR013) \| 99% \| 98.78 \| \| Sarcocystis sp. clone Ma6.5 \| [OL830303.1](https://www.ncbi.nlm.nih.gov/nucleotide/OL830303.1?report=genbank&log$=nucltop&blast_rank=15&RID=XUT9F8KR013) \| 99% \| 98.78 \| \| Sarcocystis sp. clone Ma37.4 \| [OL830329.1](https://www.ncbi.nlm.nih.gov/nucleotide/OL830329.1?report=genbank&log$=nucltop&blast_rank=16&RID=XUT9F8KR013) \| 99% \| 98.48 \| \| Sarcocystis sp. clone Ma36.2 \| [OL830325.1](https://www.ncbi.nlm.nih.gov/nucleotide/OL830325.1?report=genbank&log$=nucltop&blast_rank=17&RID=XUT9F8KR013) \| 99% \| 98.48 \| \| Sarcocystis sp. clone Ma30.1 \| [OL830323.1](https://www.ncbi.nlm.nih.gov/nucleotide/OL830323.1?report=genbank&log$=nucltop&blast_rank=18&RID=XUT9F8KR013) \| 99% \| 98.48 \| \| Sarcocystis sp. clone Ma18.5 \| [OL830314.1](https://www.ncbi.nlm.nih.gov/nucleotide/OL830314.1?report=genbank&log$=nucltop&blast_rank=19&RID=XUT9F8KR013) \| 99% \| 98.48 \| \| Sarcocystis sp. clone Ma12.4 \| [OL830313.1](https://www.ncbi.nlm.nih.gov/nucleotide/OL830313.1?report=genbank&log$=nucltop&blast_rank=20&RID=XUT9F8KR013) \| 99% \| 98.48 \| \| Sarcocystis sp. clone Ma12.1 \| [OL830311.1](https://www.ncbi.nlm.nih.gov/nucleotide/OL830311.1?report=genbank&log$=nucltop&blast_rank=21&RID=XUT9F8KR013) \| 99% \| 98.48 \| \| Sarcocystis sp. clone Ma37.3 \| [OL830328.1](https://www.ncbi.nlm.nih.gov/nucleotide/OL830328.1?report=genbank&log$=nucltop&blast_rank=22&RID=XUT9F8KR013) \| 99% \| 98.18 \| \| Sarcocystis sp. clone Ma29.2 \| [OL830322.1](https://www.ncbi.nlm.nih.gov/nucleotide/OL830322.1?report=genbank&log$=nucltop&blast_rank=23&RID=XUT9F8KR013) \| 99% \| 98.18 \| \| Sarcocystis sp. clone Ma28.4 \| [OL830320.1](https://www.ncbi.nlm.nih.gov/nucleotide/OL830320.1?report=genbank&log$=nucltop&blast_rank=24&RID=XUT9F8KR013) \| 99% \| 98.18 \| \| Sarcocystis sp. clone Ma11.1 \| [OL830308.1](https://www.ncbi.nlm.nih.gov/nucleotide/OL830308.1?report=genbank&log$=nucltop&blast_rank=25&RID=XUT9F8KR013) \| 99% \| 98.18 \| \| Sarcocystis sp. clone Ma9.4 \| [OL830307.1](https://www.ncbi.nlm.nih.gov/nucleotide/OL830307.1?report=genbank&log$=nucltop&blast_rank=26&RID=XUT9F8KR013) \| 99% \| 98.18 \| \| Sarcocystis sp. clone Ma9.3 \| [OL830306.1](https://www.ncbi.nlm.nih.gov/nucleotide/OL830306.1?report=genbank&log$=nucltop&blast_rank=27&RID=XUT9F8KR013) \| 99% \| 98.18 \| \| Sarcocystis sp. clone Ma9.2 \| [OL830305.1](https://www.ncbi.nlm.nih.gov/nucleotide/OL830305.1?report=genbank&log$=nucltop&blast_rank=28&RID=XUT9F8KR013) \| 99% \| 98.18 \| \| Sarcocystis falcatula isolate Lorikeet ID #205850 \| [MH626538.1](https://www.ncbi.nlm.nih.gov/nucleotide/MH626538.1?report=genbank&log$=nucltop&blast_rank=29&RID=XUT9F8KR013) \| 96% \| 98.76 \| \| Sarcocystis sp. clone Ma37.1 \| [OL830327.1](https://www.ncbi.nlm.nih.gov/nucleotide/OL830327.1?report=genbank&log$=nucltop&blast_rank=30&RID=XUT9F8KR013) \| 99% \| 97.87 \| \| Sarcocystis lindsayi \| [AF387164.1](https://www.ncbi.nlm.nih.gov/nucleotide/AF387164.1?report=genbank&log$=nucltop&blast_rank=31&RID=XUT9F8KR013) \| 100% \| 93.43 \| \| Sarcocystis neurona \| [AF204230.1](https://www.ncbi.nlm.nih.gov/nucleotide/AF204230.1?report=genbank&log$=nucltop&blast_rank=32&RID=XUT9F8KR013) \| 83% \| 99.28 \| \| Sarcocystis neurona \| [AH009986.2](https://www.ncbi.nlm.nih.gov/nucleotide/AH009986.2?report=genbank&log$=nucltop&blast_rank=33&RID=XUT9F8KR013) \| 79% \| 100 \| |  |  |  |
| --- | --- | --- | --- | --- | --- | --- | --- | --- | --- | --- | --- | --- | --- | --- | --- | --- | --- | --- | --- | --- | --- | --- | --- | --- | --- | --- | --- | --- | --- | --- | --- | --- | --- | --- | --- | --- | --- | --- | --- | --- | --- | --- | --- | --- | --- | --- | --- | --- | --- | --- | --- | --- | --- | --- | --- | --- | --- | --- | --- | --- | --- | --- | --- | --- | --- | --- | --- | --- | --- | --- | --- | --- | --- | --- | --- | --- | --- | --- | --- | --- | --- | --- | --- | --- | --- | --- | --- | --- | --- | --- | --- | --- | --- | --- | --- | --- | --- | --- | --- | --- | --- | --- | --- | --- | --- | --- | --- | --- | --- | --- | --- | --- | --- | --- | --- | --- | --- | --- | --- | --- | --- | --- | --- | --- | --- | --- | --- | --- | --- | --- | --- | --- | --- | --- | --- | --- | --- | --- | --- |
