# Supplementary material for: Case report: Sarcocystis speeri, Aspergillus fumigatus, and novel Treponema sp. infections in an adult Atlantic spotted dolphin (Stenella frontalis)
Source: Front Vet Sci. 2023 Apr 3;10:1132161. doi: 10.3389/fvets.2023.1132161 (PMC10106728; doi:10.3389/fvets.2023.1132161)
Supplement: Supplementary file 3 [file Table_3.docx]

| Table S3. BLASTN Results for apicomplexan ITS sequence amplified from frozen CNS samples of an Atlantic spotted dolphin (*Stenella frontalis*)   \| Description \| Accession \| Query Coverage \| % identity \| \| --- \| --- \| --- \| --- \| \| Sarcocystis speeri \| [KT207458.1](https://www.ncbi.nlm.nih.gov/nucleotide/KT207458.1?report=genbank&log$=nucltop&blast_rank=1&RID=XUU124K7013) \| 100% \| 100 \| \| Sarcocystis falcatula isolate Lorikeet ID #205850 \| [MH626538.1](https://www.ncbi.nlm.nih.gov/nucleotide/MH626538.1?report=genbank&log$=nucltop&blast_rank=2&RID=XUU124K7013) \| 100% \| 97.18 \| \| Sarcocystis falcatula strain Florida 1 \| [AF098244.1](https://www.ncbi.nlm.nih.gov/nucleotide/AF098244.1?report=genbank&log$=nucltop&blast_rank=3&RID=XUU124K7013) \| 94% \| 97.02 \| \| Sarcocystis neurona strain UCD 2 \| [AH008471.2](https://www.ncbi.nlm.nih.gov/nucleotide/AH008471.2?report=genbank&log$=nucltop&blast_rank=4&RID=XUU124K7013) \| 87% \| 99.13 \| \| Sarcocystis neurona \| [AH009986.2](https://www.ncbi.nlm.nih.gov/nucleotide/AH009986.2?report=genbank&log$=nucltop&blast_rank=5&RID=XUU124K7013) \| 85% \| 99.4 \| \| Sarcocystis neurona \| [AF252407.1](https://www.ncbi.nlm.nih.gov/nucleotide/AF252407.1?report=genbank&log$=nucltop&blast_rank=6&RID=XUU124K7013) \| 85% \| 99.4 \| \| Sarcocystis neurona isolate UCD1 \| [AY082644.1](https://www.ncbi.nlm.nih.gov/nucleotide/AY082644.1?report=genbank&log$=nucltop&blast_rank=7&RID=XUU124K7013) \| 84% \| 99.7 \| \| Sarcocystis dasypi clone 219 \| [AY082633.1](https://www.ncbi.nlm.nih.gov/nucleotide/AY082633.1?report=genbank&log$=nucltop&blast_rank=8&RID=XUU124K7013) \| 84% \| 99.7 \| \| Sarcocystis dasypi clone 217 \| [AY082631.1](https://www.ncbi.nlm.nih.gov/nucleotide/AY082631.1?report=genbank&log$=nucltop&blast_rank=9&RID=XUU124K7013) \| 84% \| 99.7 \| \| Sarcocystis neurona \| [AF081944.2](https://www.ncbi.nlm.nih.gov/nucleotide/AF081944.2?report=genbank&log$=nucltop&blast_rank=10&RID=XUU124K7013) \| 85% \| 98.8 \| \| Sarcocystis neurona \| [AY082637.1](https://www.ncbi.nlm.nih.gov/nucleotide/AY082637.1?report=genbank&log$=nucltop&blast_rank=11&RID=XUU124K7013) \| 84% \| 99.39 \| \| Sarcocystis neurona \| [AY082636.1](https://www.ncbi.nlm.nih.gov/nucleotide/AY082636.1?report=genbank&log$=nucltop&blast_rank=12&RID=XUU124K7013) \| 84% \| 99.09 \| \| Sarcocystis neurona \| [AY082634.1](https://www.ncbi.nlm.nih.gov/nucleotide/AY082634.1?report=genbank&log$=nucltop&blast_rank=13&RID=XUU124K7013) \| 84% \| 99.09 \| \| Sarcocystis falcatula \| [AF098246.1](https://www.ncbi.nlm.nih.gov/nucleotide/AF098246.1?report=genbank&log$=nucltop&blast_rank=14&RID=XUU124K7013) \| 94% \| 95.38 \| \| Sarcocystis neurona \| [AY082648.1](https://www.ncbi.nlm.nih.gov/nucleotide/AY082648.1?report=genbank&log$=nucltop&blast_rank=15&RID=XUU124K7013) \| 84% \| 99.09 \| \| Sarcocystis neurona \| [AY082635.1](https://www.ncbi.nlm.nih.gov/nucleotide/AY082635.1?report=genbank&log$=nucltop&blast_rank=16&RID=XUU124K7013) \| 84% \| 99.09 \| \| Sarcocystis falcatula \| [MW822673.1](https://www.ncbi.nlm.nih.gov/nucleotide/MW822673.1?report=genbank&log$=nucltop&blast_rank=17&RID=XUU124K7013) \| 84% \| 98.17 \| \| Sarcocystis falcatula strain Cornell 1 \| [AF098242.1](https://www.ncbi.nlm.nih.gov/nucleotide/AF098242.1?report=genbank&log$=nucltop&blast_rank=18&RID=XUU124K7013) \| 86% \| 97.02 \| \| Sarcocystis neurona \| [AF204230.1](https://www.ncbi.nlm.nih.gov/nucleotide/AF204230.1?report=genbank&log$=nucltop&blast_rank=19&RID=XUU124K7013) \| 80% \| 99.04 \| \| Sarcocystis sp. AGP-1 \| [DQ768306.1](https://www.ncbi.nlm.nih.gov/nucleotide/DQ768306.1?report=genbank&log$=nucltop&blast_rank=20&RID=XUU124K7013) \| 92% \| 92.74 \| \| Sarcocystis lindsayi \| [AF387164.1](https://www.ncbi.nlm.nih.gov/nucleotide/AF387164.1?report=genbank&log$=nucltop&blast_rank=21&RID=XUU124K7013) \| 91% \| 93.42 \| \| Sarcocystis falcatula isolate MP20 \| [MW822672.1](https://www.ncbi.nlm.nih.gov/nucleotide/MW822672.1?report=genbank&log$=nucltop&blast_rank=22&RID=XUU124K7013) \| 84% \| 96.65 \| |  |  |  |
| --- | --- | --- | --- | --- | --- | --- | --- | --- | --- | --- | --- | --- | --- | --- | --- | --- | --- | --- | --- | --- | --- | --- | --- | --- | --- | --- | --- | --- | --- | --- | --- | --- | --- | --- | --- | --- | --- | --- | --- | --- | --- | --- | --- | --- | --- | --- | --- | --- | --- | --- | --- | --- | --- | --- | --- | --- | --- | --- | --- | --- | --- | --- | --- | --- | --- | --- | --- | --- | --- | --- | --- | --- | --- | --- | --- | --- | --- | --- | --- | --- | --- | --- | --- | --- | --- | --- | --- | --- | --- | --- | --- | --- | --- | --- | --- |
